# Off like a shot: scaling of ballistic tongue projection reveals extremely high performance in small chameleons

**DOI:** 10.1038/srep18625

**Published:** 2016-01-04

**Authors:** Christopher V. Anderson

**Affiliations:** 1 Department of Ecology and Evolutionary Biology, Brown University, Providence, RI 02912, USA

## Abstract

Stretching elastic tissues and using their recoil to power movement allows organisms to release energy more rapidly than by muscle contraction directly, thus amplifying power output. Chameleons employ such a mechanism to ballistically project their tongue up to two body lengths, achieving power outputs nearly three times greater than those possible *via* muscle contraction. Additionally, small organisms tend to be capable of greater performance than larger species performing similar movements. To test the hypothesis that small chameleon species outperform larger species during ballistic tongue projection, performance was examined during feeding among 20 chameleon species in nine genera. This revealed that small species project their tongues proportionately further than large species, achieving projection distances of 2.5 body lengths. Furthermore, feedings with peak accelerations of 2,590 m s^−2^, or 264 *g*, and peak power output values of 14,040 W kg^−1^ are reported. These values represent the highest accelerations and power outputs reported for any amniote movement, highlighting the previously underestimated performance capability of the family. These findings show that examining movements in smaller animals may expose movements harbouring cryptic power amplification mechanisms and illustrate how varying metabolic demands may help drive morphological evolution.

Rapid recoil of elastic tissues enables organisms to achieve power outputs that exceed the maximum power capacities of muscle^1^,^2^. In systems employing such a mechanism, muscle contraction stretches elastic elements prior to the onset of movement, loading them with potential energy. When released, the recoil of these elastic tissues releases stored energy more rapidly than the muscles are capable of producing, providing high power to associated movements^2^,^3^. Such power amplification is particularly useful in highly dynamic movements with short accelerative phases, such as jumping, which necessitate the rapid release of energy to maximize performance^1^,^3^,^4^,^5^,^6^.

Small organisms often exhibit higher locomotor performance for their body size than their larger relatives^4^,^7^,^8^,^9^. For example, jump distance is a function of takeoff velocity^4^,^9^, which is said to be size independent among geometrically similar animals^7^. Therefore, geometrically similar animals should jump the same absolute height, regardless of their body size^4^,^8^,^9^. That height, however, is considerably larger relative to body length in smaller organisms. Further, smaller organisms have a shorter distance over which to reach the same takeoff velocity as larger animals, due to their shorter limbs, and therefore must produce significantly higher acceleration than larger animals^4^,^9^. As a result, small animals must release a proportional amount of energy over a shorter accelerative period^4^,^9^, resulting in higher mass-specific power output values. Thus, the relationship between changes in body size, morphological proportions and performance capacity directly affects how organisms function in their natural environment^4^,^8^,^9^. As body size varies through ontogeny or among species of differing size, many animals display shifts in their morphological proportions, whole-organism performance, behaviour, or ecology as a result of functional tradeoffs associated with these changes^7^,^10^,^11^,^12^. Understanding these proximate relationships can therefore aid our understanding of organism function, ecology and evolution.

Chameleons employ a power amplification mechanism to ballistically project their tongue as far as two body lengths from their mouth to capture prey^13^,^14^,^15^,^16^,^17^,^18^. To do so, the tongue is rapidly accelerated off the hyoid – achieving accelerations as high as 486 m s^−2^ (50 *g*)^19^ – with the tongue subsequently traveling to the prey on its momentum alone^15^. In the process, the peak muscle mass-specific power that would be required to generate the observed accelerations reach as high as 3,185 W kg^−1^ 20. This performance exceeds that for which vertebrate muscle is known to produce *in vivo* – estimated at 1,121 W kg^−1^ during vertical takeoff in flying quail^21^ – by almost three times.

Most studies of chameleon tongue projection have examined chameleon species that exceed 100 mm in snout-vent length (SVL)^13^,^14^,^17^,^18^,^19^,^20^,^22^,^23^,^24^,^25^. Recent anatomical work, however, has found that both within and among species, smaller chameleons have a proportionately larger tongue apparatus than larger chameleons^26^,^27^ and thus may have relatively longer tongue projection distances. Similarly, variation in body and tongue apparatus size might result in variation in other performance parameters, such as projectile velocity, acceleration, and power output. This variation could mean that some of the most impressive statistics on chameleon feeding performance have been underestimated due to sampling bias, and more importantly that our understanding of how variation in the ecology and physiology of these animals relates to their organismal performance may be incomplete.

Using phylogenetic comparative methods, maximal feeding performance among 20 chameleon taxa spanning a five-fold range of adult body size was examined to test scaling hypotheses stemming from previous morphological examination of the tongue apparatus^26^. In particular, I hypothesized that (i) because the mass of the tongue retractor muscle is known to scale with negative allometry relative to SVL among chameleon species^26^, tongue projection distance will scale with negative allometry relative to SVL as well (i.e., smaller species are expected to project their tongues proportionately longer distances than larger species); (ii) peak tongue projection velocity will scale with isometry relative to SVL, with velocity being size independent; (iii) peak tongue projection acceleration and peak muscle mass-specific power will both decrease with increasing body size; and (iv) because the chameleon tongue apparatus scales with negative allometry relative to SVL^26^, peak acceleration and peak mass-specific power will both scale with positive allometry relative to SVL (i.e., acceleration and power are expected to be proportionately lower at smaller body size than expected based on geometric similarity).

## Results

**Figure 1:**
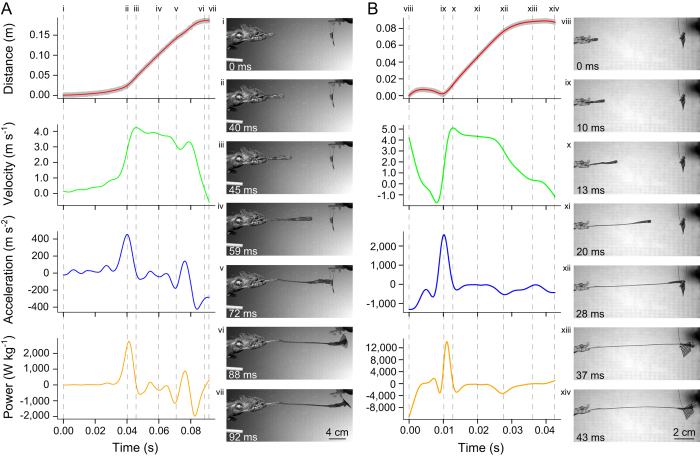
Kinematic and dynamic profiles, and associated image sequences comparing a tongue projection event in (**A**) a 198 mm SVL *Furcifer oustaleti* exhibiting relatively low maximal performance and (**B**) a 47 mm SVL *Rhampholeon spinosus* exhibiting particularly high maximal performance. The feeding from *F. oustaleti* exhibits maximal performance levels consistent with previously published values for a similar sized *Furcifer* species: average of 2,340 W kg^−1^ (s.d. = 352 W kg^−1^; *n* = 13) in a 180 mm SVL *F. pardalis*^14^. The feeding from *R. spinosus* represents the highest recorded acceleration and power values recorded in our study. Vertical dashed lines correspond to the timing of images at right, corresponding to (i, viii) the start of the analyzed sequence, (ii, ix) the time of peak acceleration, (iii, x) the time of peak velocity, (iv, xi) an intermediate point during tongue projection, (v, xii) the point of prey contact, (vi, xiii) the time of maximal tongue projection distance, and (vii, xiv) the end of the analyzed sequence. Distance traces show raw position data (gray) with smoothed position trace (red) overlaid. Subsequent velocity, acceleration and power traces are calculated from the smoothed position trace.

In total, 279 feeding events from 55 different individuals were analyzed. This sample represented 20 chameleon species in nine genera (Table 1 and Supplementary Fig. S1), covering approximately 10% of the species-level and 75% of the genus-level diversity of the family^28^. Individuals ranged in SVL from 40 to 198 mm (Table 1), representing an approximate five-fold range in SVL. Maximal tongue projection distances ranged from 37.0 to 267.6 mm; when these distances were adjusted for body size, tongue projection length averaged 1.5 times the SVL, but reached as high as 2.5 times SVL (Supplementary Table S1). The maximum peak projection velocity achieved ranged from 2.91 to 5.41 m s^−1^, while maximum peak acceleration ranged from 286 to 2,590 m s^−2^, or 29 to 264 *g* (Fig. 1 and Table 1). Further, the maximum peak muscle mass-specific power that would be required to generate the observed accelerations ranged from 1,410 to 14,040 W kg^−1^.

**Figure 2:**
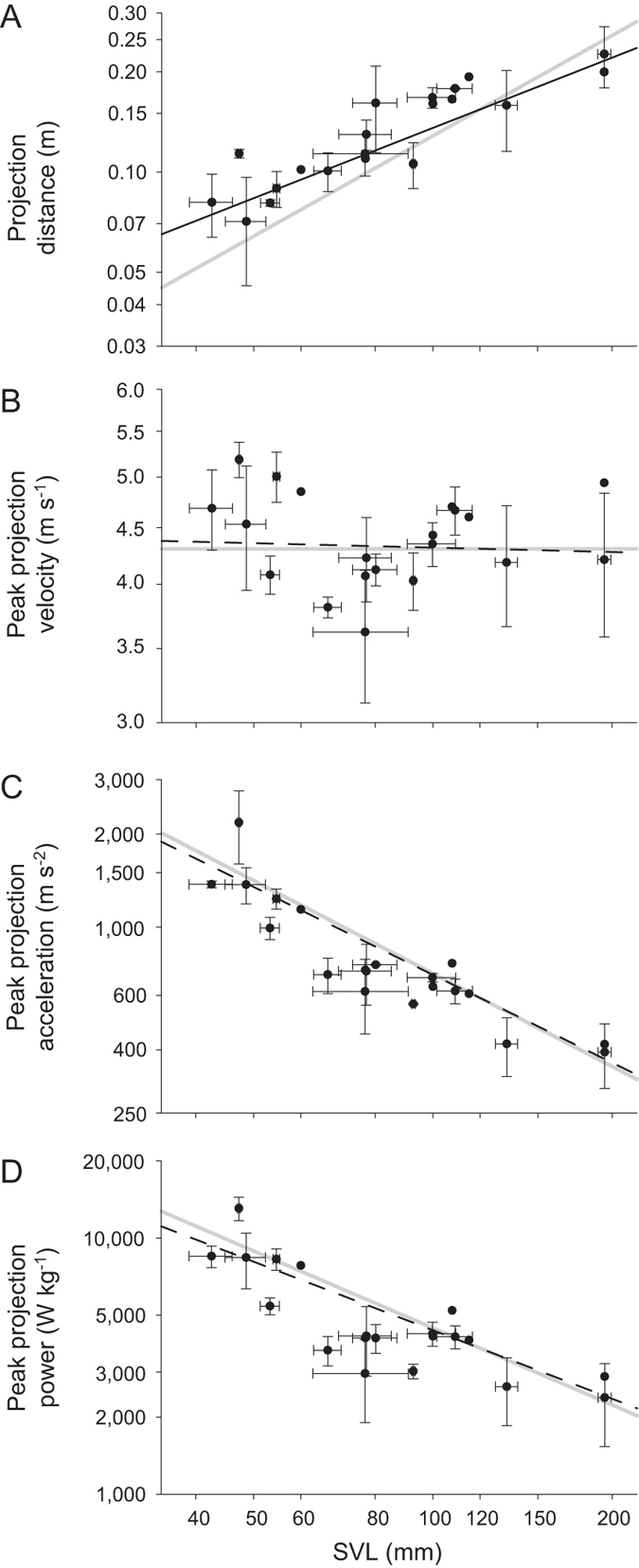
Scaling relationships among species for (**A**) peak projection distance, (**B**) peak projection velocity, (**C**) peak projection acceleration and (**D**) peak mass-specific power output with respect to SVL. Graphs depict raw species averages and standard deviations of maximal performance for individuals on log axes. Solid light gray lines represent isometric slope. Dark gray lines represent observed scaling relationships among species. Solid dark gray lines represent observed scaling relationships significantly different from that expected by isometry (i.e., expected slope falls outside the 95% confidence interval around the observed slope). Dashed dark gray lines represent observed scaling relationships not significantly different from that expected by isometry.

Jaw length scaled with negative allometry (observed slope lower than predicted by isometry) relative to SVL, with the jaw being proportionately longer in small species than in large species (Table 2). Despite this allometry, however, each performance variable scaled in a consistent way relative to both jaw length and SVL (Table 2, Fig. 2 and Supplementary Fig. S2). Specifically, projection distance and projection duration each scaled with negative allometry relative to both SVL and jaw length. Peak tongue projection velocity, peak acceleration and peak muscle mass-specific power, on the other hand, all scaled with isometry relative to both SVL and jaw length.

## Discussion

To date, most studies quantifying tongue projection performance in chameleons have used species larger than 100 mm SVL^13^,^14^,^17^,^18^,^19^,^20^,^22^,^23^,^24^,^25^. Chameleons of this size are often more readily available, hardier captives, and produce larger movements, making them easier for experimental and observational work. Data in this study examining scaling patterns of feeding performance show, however, that the use of larger species underestimates peak performance of the family due to both typical scaling effects as predicted by geometric similarity and morphological proportions that exhibit allometric scaling patterns. For instance, the mass of the tongue retractor muscle in chameleons scales with negative allometry relative to body length among species^26^, and this study confirms that smaller chameleon species are similarly able to project their tongues proportionately longer distances than larger species. Previously published studies documented chameleons as capable of achieving tongue projection distances as high as two body lengths^13^,^14^,^16^,^17^,^18^. In this study, however, tongue projection distances as high as 2.5 body lengths in a 47 mm SVL specimen of *Rhampholeon spinosus* were recorded. Similarly, tongue projection distances longer than 2 body lengths were also recorded in *Brookesia superciliaris, Rieppeleon brevicaudatus* and *Trioceros hoehnelii*, all of which are smaller than 90 mm SVL. Further, consistent with scaling relationships based on geometric similarity^7^,^8^,^9^,^26^, this study found that both acceleration and mass-specific power increased as body size decreased. Previous studies using chameleons that exceeded 220 mm SVL documented chameleons projecting their tongues with peak accelerations of up to 486 m s^−2^, or almost 50 *g*^19^, and peak mass-specific power output values of 3,185 W kg^−1^ 20. Here, however, I show peak accelerations as high as 2,590 m s^−2^, or 264 *g*, and peak muscle mass-specific power values up to 14,040 W kg^−1^ in a 47 mm SVL individual of *Rhampholeon spinosus*.

Examples of extreme performance are well known among other small organisms, particularly small invertebrates. Some planthoppers, for example, jump with accelerations as high as 7,051 m s^−2^ (719 *g*) and power output values as high as 160,300 W kg^−1^ 29. Similarly, mantis shrimp have been reported to produce predatory strikes with an acceleration of 104,000 m s^−2^ (10,605 *g*) and power output values as high as 470,000 W kg^−1^ 30, and trap-jaw ants snap their jaws closed at accelerations on the order of 10^5^ × *g*^31^. Among vertebrates, extreme movements produce performance levels that are also impressive, albeit more modest than those seen in invertebrates. For example, toads have been recorded projecting their tongue with accelerations as high as 1,440 m s^−2^ (147 *g*)^32^ and power output values as high as 9,600 W kg^−1^ 33. Plethodontid salamanders, on the other hand, project their tongues at accelerations up to 4,492 m s^−2^ (458 *g*) and power output values as high as 18,129 W kg^−1^ 34. Among amniotes, however, performance is relatively reduced, even among movements powered by elastic recoil. Jumping in bushbabies, for instance, has been estimated to require power outputs of 1,700–2,350 W kg^−1^ of muscle mass^5^ at recorded accelerations of up to 120 m s^−2^ (12 *g*)^35^. Assuming the same proportional mass of the muscles involved in jumping relative to body mass reported in bushbabies (40%)^5^, jumps in tarsiers would require 1820 W kg^−1^ 35. Finally, although not powered by elastic recoil, striking in vipers can achieve accelerations as high as 119 m s^−2^ (12 *g*)^36^, and neck extension in snapping turtles can achieve over 160 m s^−2^ (16 *g*)^37^. While the performance levels recorded in this study do not approach those for many movements in invertebrates, our data show that chameleons are able to produce peak accelerations and peak mass specific power values during tongue projection that are the highest among any amniote movement.

Extreme performance like that observed in chameleons is often used as an indicator for the presence of a power amplification mechanism. Such mechanisms are indicated when the peak mass-specific power output required to produce the accelerations associated with an observed movement exceeds that for which muscle is known to produce *in vivo*. This logic has been used to implicate power amplification mechanisms in various systems, including the jumping of insects^29^,^38^ and frogs^1^, and tongue projection in salamanders^34^, toads^33^ and chameleons^20^. Similar elastic recoil mechanisms may be used, however, even when calculated power output levels fail to exceed known maxima for muscle^2^. My results suggest that because of observed scaling patterns for power, the presence of power amplification mechanisms may be clearly demonstrated by examining small species, and likely small individuals within a species, where the examination of larger species or individuals might have otherwise been inconclusive.

The scaling of performance may similarly shed light on patterns of morphological evolution. For instance, previous studies have proposed two alternative hypotheses to explain why small chameleon species have a proportionately larger tongue apparatus than larger species^26^. First, given the higher mass-specific metabolic rates of smaller animals^39^,^40^,^41^,^42^,^43^, small chameleon species may be under pressure to increase the effectiveness of their feeding apparatus in order to mitigate metabolic scaling constraints^26^. Under this scenario, one would expect small chameleons to project their tongues proportionately further than large species and be capable of capturing larger prey. Alternatively, because internal deformations of soft tissue can result in significant energy loss^44^, high accelerations may be unfavorable for small, soft tissue projectiles^44^,^26^. This explanation would suggest that accelerations associated with maximal tongue projection performance should be relatively lower than expected by body length alone in small species, thereby potentially reducing energy loss during tongue projection. This study found, however, that both peak acceleration and power output scaled in direct proportion to body size, suggesting that the energy potentially lost due to high accelerations is not reduced in small species by having proportionately lower accelerations for their body size. On the other hand, with proportionately longer jaws, a proportionately larger tongue apparatus^26^, proportionately larger tongue muscle cross sectional areas^27^, and a proportionately longer tongue projection distance relative to their body length, small chameleons have effectively increased the relative size of their entire feeding apparatus. In doing so, small chameleons have increased the functional range of their prey capture mechanism, and are likely able to capture and process larger prey items than they would otherwise be able to if their muscle cross sections and jaws were not disproportionately large for their body size. This inference is supported by the selection of proportionately larger prey items by the smaller of two morphological forms in *Bradypodion*^45^. These patterns are thus consistent with those that would be predicted for mitigating metabolic scaling constraints, which may be involved in driving the observed morphological scaling patterns.

## Conclusions

Although most previous studies on chameleon feeding have used larger species, this data reveals that small chameleon species are able to feed with more extreme performance than their larger relatives due to both typical scaling effects as predicted by geometric similarity and the relatively larger tongue apparatus of small species. In particular, small chameleons are able to project their tongues proportionately longer distances, with projection lengths reaching 2.5 body lengths. Additionally, small chameleon species are shown to be capable of producing peak accelerations during tongue projection of up to 2,590 m s^−2^, or 264 *g*, and mass-specific power output values during tongue projection of up to 14,040 W kg^−1^, values that are the highest reported among amniotes. These scaling relationships not only highlight the previously underestimated performance capability of the family, but also the potential utility of taking body size into account when testing for movements harbouring cryptic power amplification mechanisms and how varying metabolic demands may help drive morphological evolution.

## Materials and Methods

### Animals

Feeding events were recorded from 20 chameleon taxa. Individuals from these species were a mix of wild-caught and captive-bred specimens that were collected for the purposes of other studies, obtained through the pet trade, or loaned from private facilities for non-invasive feeding trials. At the time of data collection, SVL and jaw length of each individual was measured using Mitutoyo electronic calipers (±0.1 mm).

Chameleons were maintained individually or in small groups in either mesh-sided enclosures or glass terraria with live plants, depending on the species. Enclosures were equipped with fluorescent UVB lighting on a 12 h per day light cycle. Ambient temperatures were maintained between 20 and 24 °C and a basking spot of approximately 30–35 °C was provided where appropriate for the individual species. Hydration was maintained *via* bi-daily misting and individuals were fed a diet of gut-loaded crickets.

All procedures were approved by the University of South Florida Institutional Animal Care and Use Committee (W3441 and W4074 to Stephen M. Deban) and, where applicable, ethical clearance was obtained from the South African National Biodiversity Institute (002/2011 to Krystal A. Tolley). Portions of this work were carried out under permits from Ezemvelo KZN Wildlife (OP 696/2012 to C.V.A.) and CapeNature (0010-AAA004-00858 and 0011-AAA004-00405 to C.V.A.). All experiments were performed in accordance with relevant guidelines and regulations.

### Feeding experiments

All chameleons were imaged at 3 kHz with a Photron Fastcam 1024 PCI camera as they fed on crickets. Chameleons were placed on a wooden dowel oriented parallel to the image plane of the camera. Crickets were placed on a square of insect screen suspended by thread in front of the dowel to create a “cricket trapeze,” which allowed the chameleon’s tongue to complete its trajectory unimpeded (Fig. 1)^22^. Distance between the camera and wooden dowel varied based on body size such that tongue projection distance approximately filled the recording field for all body sizes. Body temperature was measured orally using a calibrated infrared thermometer (Sixth Sense LT300; ±1 °C accuracy) following every feeding event. Only feeding sequences for which body temperature fell within the 20–25 °C range were used for this study as chameleons experience minimal thermal effects across this temperature range (*Q*_10_ values of 1.1–1.2 over the 15–25 °C range)^22^,^46^. Up to ten feeding events were gathered for each individual over the course of up to two months with no more than five feedings collected on any given day.

### Kinematic and dynamic analyses

Tongue projection performance for each scale-calibrated feeding was computed using National Institutes of Health ImageJ software (http://imagej.nih.gov/ij/). To characterize performance, five variables were measured: (i) maximum tongue projection distance; (ii) tongue projection duration; (iii) peak velocity of tongue projection; (iv) peak acceleration of tongue projection; and (v) the peak muscle mass-specific power that would be required to generate the observed accelerations. Tongue projection distance was measured as the maximal distance from the tongue tip to the dentary tip during a feeding event. Projection duration was measured as the time lapsed between the onset of tongue projection, defined as the time where velocity first began a rapid increase prior to peaking at the onset of the ballistic phase of tongue projection, and the time the tongue reached maximal projection distance. The *x, y* coordinates of the tip of the tongue on each frame throughout the tongue projection sequence were recorded using ImageJ software. A quintic spline was fitted to the resultant position trace of the tongue using a custom script including the P-spline package of R statistical software (www.r-project.org) and smoothed to remove secondary oscillation artefacts from the first and second derivatives of position. This spline and smoothing method was used as this technique is unlikely to overestimate velocities and accelerations, and is generally more robust than other numerical differentiation algorithms, particularly for estimating accelerations^47^. From these smoothed position data, instantaneous velocity (m s^−1^) and acceleration (m s^−2^) traces (i.e., the first and second derivatives of the position) were calculated. Mass-specific power (W kg^−1^) was calculated as the product of velocity and acceleration, and corrected for the mass of the projector muscle. Given that the relative proportions among the musculoskeletal components within the chameleon feeding apparatus are conserved both within and among species^26^, I corrected for the mass of the projector muscle (the M. accelerator linguae) using the correction used in other studies^20^,^22^: by multiplying mass-specific power by a factor of two to obtain power in units of W per kg of muscle mass required to produce the observed movement. To estimate an individual’s maximal performance capability, only the maximal performance among trials for each individual was used in subsequent analyses.

An error resulted in a portion of the feedings recorded from the five *Bradypodion* spp. being saved at a frame rate of 1.5 kHz rather than the 3 kHz film rate. Twelve feedings from some of the same individuals were analyzed at 3 kHz and compared to down-sampled outputs of those feedings to replicate the saved 1.5 kHz frame rate. When traces were smoothed to similar levels, as is done in all videos, it was found that performance parameters on average only varied between 0.5% and 3.3%. This variation was considered negligible and as a result those feedings saved at a frame rate of 1.5 kHz were included to maximize the sample size.

To verify that kinematic profiles of feeding events were smoothed below any inherent digitizing error, a *post hoc* test was performed on a subset of feedings. Five feeding events were selected for this test that covered the complete range of acceleration performance and body size: the feedings with the highest individual performance from the (i) largest and (ii) smallest chameleon in the study, the feedings with the overall (iii) highest and (iv) lowest acceleration recorded, and (v) a feeding with intermediate overall performance in a chameleon of intermediate size. From these feedings, the position of two points of known distance from each other were digitized in each frame of the feeding sequence. The standard deviation of the calculated intermarker distance between these points, as a measure of digitizing error, was then used as the smoothing input value for each associated feeding sequence. Resultant performance traces and peak performance values were then compared to traces and values obtained during the original analyses. In all cases, the traces based on the digitizing error were less smoothed and resulted in significantly higher peak performance values than those of the original analysis, supporting that these feeding sequences had been smoothed below any inherent digitizing error band.

### Expected scaling relationships

Expected scaling relationships between body size measures and performance traits were based on predictions of an isometric scaling relationship (i.e., geometric similarity)^7^,^8^,^9^. Under these predictions morphological lengths should scale in direct proportion to each other (i.e., slope = 1.0), movement velocities should be size independent (i.e., slope = 0.0), and movement accelerations should be inversely proportional to length (i.e., slope = −1.0). Additionally, as velocity is size independent and lengths scale in direct proportion to each other, movement durations would be expected to scale in proportion to length (i.e., slope = 1.0). Further, although the total power output a muscle is able to produce is predicted to scale with mass to the 2/3 power (i.e., length to the power of 2)^9^, mass-specific power would scale as the scaling relationship of power output divided by mass, which is equal to mass to the −1/3 power (i.e., length to the power of −1). Thus, mass-specific power would be predicted to be inversely proportional to length (i.e., slope = −1.0). Finally, since the length of the tongue retractor muscle, the m. hyoglossus, limits tongue projection distance^15^,^26^,^48^, tongue projection distance should scale in direct proportion to morphological lengths (i.e., slope = 1.0) under geometric similarity.

### Phylogenetic analyses

To estimate the relationships among chameleon taxa for phylogenetically corrected analysis, a pruned chronogram of a tree that had been published previously^49^ was constructed. This previously published chronogram contained all taxa included in this study except for an as yet undescribed taxon, the so-called “Emerald Dwarf Chameleon”^50^ (henceforth *Bradypodion* sp. “emerald”). This taxon represents a recent divergence from *B. thamnobates*^51^,^52^,^53^,^54^, so *B.* sp. “emerald” was assigned as a sister taxon to *B. thamnobates* and assigned branch lengths close to zero (Supplementary Fig. S1).

Prior to analysis, all measurements were log transformed, allowing scaling relationships to be examined using linear regression where the scaling relationship is the coefficient of the equation for the fitted line (i.e., the slope). Regression coefficients were calculated using the Contrast program in PHYLIP, version 3.695^55^. The W menu option of this program was invoked, which calculates contrasts based on both within- and among-species covariation by including all individuals in the model rather than an average value for each species^56^. From the covariances, correlations and regressions given in the output files, 95% confidence limits around the regression coefficients were calculated based on the mathematical relationship between the standard error of the regression coefficient and the covariance, correlation coefficient and regression coefficient^26^,^57^,^58^. Isometry was rejected if the expected regression coefficient did not fall within the 95% confidence limits of the observed regression coefficient.

## Additional Information

**How to cite this article**: Anderson, C. V. Off like a shot: scaling of ballistic tongue projection reveals extremely high performance in small chameleons. *Sci. Rep.* **6**, 18625; doi: 10.1038/srep18625 (2016).

## Supplementary Material

Supplementary Information

## Figures and Tables

**Table 1:** Minimum and maximum values of kinematic variables.

Species	Sample size	Min./max. SVL (mm)	Min./max. Jaw length (mm)	Min./max. Max. projection distance (mm)	Min./max. Min. projection duration (ms)	Min./max. Max. peak velocity (m s^−1^)	Min./max. Max. peak acceleration (m s^−2^)	Min./max. Max. peak muscle mass-specific power (W kg^−1^)
*Bradypodion melanocephalum*	4 (17; 2–5)	51/55	11.7/12.4	78.8/83.2	19.3/23.3	3.92/4.29	920/1,100	4,920/5,840
*B. occidentale*	2 (10; 5–5)	92/93	20.6/21.7	94.1/117.6	25.3/28.0	3.86/4.20	561/565	2,880/3,160
*B. pumilum*	4 (18; 3–6)	63/70	12.9/17.2	83.6/116.5	26.7/34.7	3.74/3.91	589/813	3,020/4,140
*B.* sp. “emerald”	5 (35; 5–10)	69/88	16.3/18.7	111.1/146.7	29.0/34.0	3.66/4.59	518/944	2,600/5,720
*B. thamnobates*	6 (35; 5–10)	53/96	13.3/20.1	97.1/141.6	20.0/38.7	2.91/4.27	490/907	1,802/4,680
*Brookesia superciliaris*	2 (6; 1–5)	40/45	10.3/11.8	68.8/93.5	17.7/19.7	4.41/4.96	1,350/1,400	7,920/9,080
*Calumma p. parsonii*	1 (8)	194	43.7	199.7	39.0	4.94	417	2,880
*Chamaeleo calyptratus*	5 (45; 5–10)	125/140	30.0/34.1	101.0/195.8	26.3/39.3	3.43/4.61	305/514	1,612/3,480
*Furcifer lateralis*	1 (2)	77	20.2	109.8	32.3	4.07	726	4,080
*F. oustaleti*	3 (13; 3–5)	189/198	43.5/49.3	175.0/267.6	47.3/54.6	3.57/4.82	286/453	1,410/2,980
*Kinyongia fischeri*	2 (9; 4–5)	103/114	23.3/24.2	177.2/179.0	37.0/41.3	4.50/4.83	580/661	3,820/4,420
*K. tenuis*	1 (8)	60	15.3	101.7	12.3	4.85	1,140	7,820
*Rhampholeon acuminatus*	2 (4; 1–3)	54/55	11.8/12.0	81.5/97.1	22.7/23.3	4.82/5.19	1,170/1,300	7,720/8,840
*R. spinosus*	2 (12; 4–8)	47/47	11.6/11.9	111.3/116.0	18.3/22.7	5.05/5.32	1,770/2,590	12,100/14,040
*Rieppeleon brevicaudatus*	7 (17; 1–6)	45/55	11.5/14.2	37.0/101.0	9.7/18.7	3.60/5.41	1,090/1,620	5,120/11,620
*Trioceros cristatus*	1 (10)	108	24.3	165.5	36.7	4.70	763	5,220
*T. hoehnelii*	3 (12; 2–7)	75/88	17.9/23.0	110.7/203.3	31.0/48.3	4.03/4.28	751/757	3,480/4,500
*T. jacksonii xantholophus*	1 (2)	100	24.5	160.5	34.0	4.43	641	4,140
*T. johnstoni*	1 (3)	115	25.2	193.0	47.7	4.60	608	4,000
*T. montium*	2 (13; 5–8)	93/107	20.8/23.7	159.0/175.8	33.3/39.3	4.21/4.49	670/701	3,920/4,560

The total number of individuals is presented for each species, as well as the total number of feedings gathered from each species and the range of feedings per individual (in parentheses separated by a semicolon) in the sample size column.

Reported values represent the range of individual maximal performance for each species.

**Table 2:** Scaling relationships for kinematic measurements relative to both snout-vent length (SVL) and jaw length.

Function	Expected slope	Observed slope ± 95% confidence interval
Jaw length vs. SVL	1	0.94 ± 0.03^a^
Projection distance vs. SVL	1	0.70 ± 0.07^a^
Projection duration vs. SVL	1	0.67 ± 0.11^a^
Peak velocity vs. SVL	0	−0.01 ± 0.03
Peak acceleration vs. SVL	−1	−0.95 ± 0.08
Peak muscle mass-specific Power vs. SVL	−1	−0.89 ± 0.20
Projection distance vs. jaw length	1	0.72 ± 0.08^a^
Projection duration vs. jaw length	1	0.67 ± 0.14^a^
Peak velocity vs. jaw length	0	−0.01 ± 0.03
Peak acceleration vs. jaw length	−1	−0.99 ± 0.09
Peak muscle mass-specific Power vs. jaw length	−1	−0.92 ± 0.11

Expected slopes based on assumption of geometric similarity. Calculation of specific values are described in the Materials and methods.

^a^Expected slope falls outside the 95% confidence interval around the observed slope indicating significant difference.
