# Supplementary material for: Off like a shot: scaling of ballistic tongue projection reveals extremely high performance in small chameleons
Source: Sci Rep. 2016 Jan 4;6:18625. doi: 10.1038/srep18625 (PMC4698635; doi:10.1038/srep18625)
Supplement: Supplementary Information [file srep18625-s1.pdf]

20 Oct 2015 **Scientific Reports**

Off like a shot: scaling of ballistic tongue projection reveals extremely high performance in small chameleons

Christopher V. Anderson*

SUPPLEMENTARY MATERIALS

Department of Ecology and Evolutionary Biology, Brown University, Providence, RI 02912, USA

*Author for correspondence: Department of Ecology and Evolutionary Biology, Brown University, Providence, RI 02912, USA

(Christopher_V_Anderson@Brown.edu)

Supplemental Table S1. Summary of maximal performance recorded for feedings from each individual included in study.

Species	Individual	Sex	*n*	SVL (mm)	Jaw length (mm)	Max. projection distance (mm)	Min. projection duration (ms)	Max. peak velocity ($m\ s^{-1}$)	Max. peak acceleration ($m\ s^{-2}$)	Max. peak muscle mass-specific power ($W\ kg^{-1}$)
Bradypodion melanocephalum	Bmelo01	♂	5	51	11.7	80.6	19.3	4.12	1,010	5,680
Bradypodion melanocephalum	Bmelo05	♂	2	55	12.4	83.2	23.3	3.92	940	5,280
Bradypodion melanocephalum	Bmelo06	♂	5	54	12.0	78.8	26.7	3.99	1,100	5,840
Bradypodion melanocephalum	Bmelo09	♂	5	53	12.4	80.5	24.7	4.29	920	4,920
Bradypodion occidentale	Bocci04	♀	5	93	21.7	117.6	28.0	4.20	561	3,160
Bradypodion occidentale	Bocci08	♀	5	92	20.6	94.1	25.3	3.86	565	2,880
Bradypodion pumilum	Bpum03	♂	3	69	13.4	101.3	34.7	3.91	716	3,860
Bradypodion pumilum	Bpum04	♀	5	64	14.9	101.9	30.0	3.74	589	3,020
Bradypodion pumilum	Bpum06	♀	4	63	12.9	83.6	27.3	3.75	813	4,140
Bradypodion pumilum	Bpum10	♂	6	70	17.2	116.5	26.7	3.85	686	3,580
Bradypodion sp. “emerald”	Bsp01	♀	5	82	18.4	123.5	40.0	3.66	518	2,600
Bradypodion sp. “emerald”	Bsp05	♀	10	69	16.8	129.9	32.7	4.59	786	5,100
Bradypodion sp. “emerald”	Bsp06	♂	5	70	16.3	111.1	29.0	4.51	944	5,720
Bradypodion sp. “emerald”	Bsp07	♀	10	77	16.4	137.4	34.0	4.11	715	3,900
Bradypodion sp. “emerald”	Bsp08	♂	5	88	18.7	146.7	33.3	4.26	633	3,420
Bradypodion thamnobates	Btham01	♀	10	96	20.1	141.6	33.3	3.84	549	2,940
Bradypodion thamnobates	Btham03	♀	5	79	19.1	108.9	38.7	3.36	498	2,240
Bradypodion thamnobates	Btham05	♂	5	53	13.3	103.5	24.7	4.27	907	4,680
Bradypodion thamnobates	Btham06	♀	5	77	16.9	97.2	20.0	3.37	529	2,440
Bradypodion thamnobates	Btham09	♂	5	73	17.6	122.8	30.7	3.98	736	3,660
Bradypodion thamnobates	Btham10	♀	5	83	18.2	107.2	37.3	2.91	490	1,802
Brookesia superciliaris	superciliaris01	♀	5	45	11.8	93.5	19.7	4.96	1,400	9,080
Brookesia superciliaris	superciliaris02	♂	1	40	10.3	68.8	17.7	4.41	1,350	7,920
Calumma p. parsonii	parsonii01	♀	8	194	43.7	199.7	39.0	4.94	417	2,880
Chamaeleo calyptratus	calyptratus01	♂	10	135	32.9	195.8	30.3	4.59	514	3,480
Chamaeleo calyptratus	calyptratus02	♂	10	135	31.1	123.9	26.3	3.85	363	2,100
Chamaeleo calyptratus	calyptratus03	♂	10	140	34.1	182.8	39.3	4.45	406	2,720
Chamaeleo calyptratus	calyptratus04	♂	5	140	30.0	101.0	31.7	3.43	305	1,612

Chamaeleo calyptratus	calyptratus05	♂	10	125	30.9	189.6	31.3	4.61	503	3,240
Furcifer lateralis	lateralis01	♀	2	77	20.2	109.8	32.3	4.07	726	4,080
Furcifer oustaleti	oustaleti03	♂	5	189	49.0	267.6	54.3	4.82	440	2,980
Furcifer oustaleti	oustaleti04	♂	3	198	49.3	235.6	54.7	4.24	453	2,760
Furcifer oustaleti	oustaleti05	♀	5	196	43.5	175.0	47.3	3.57	286	1,410
Kinyongia fischeri	fischeri01	♂	4	114	24.2	179.0	37.0	4.50	661	4,420
Kinyongia fischeri	fischeri02	♂	5	104	23.3	177.2	41.3	4.83	580	3,820
Kinyongia tenuis	tenuis01	♀	8	60	15.3	101.7	12.3	4.85	1,140	7,820
Rhampholeon acuminatus	acuminatus01	♀	3	54	12.0	97.1	22.7	5.19	1,300	8,840
Rhampholeon acuminatus	acuminatus02	♀	1	55	11.8	81.5	23.3	4.82	1,170	7,720
Rhampholeon spinosus	spinosus01	♀	4	47	11.6	111.3	18.3	5.05	1,770	12,100
Rhampholeon spinosus	spinosus02	♂	8	47	11.9	116.0	22.7	5.32	2,590	14,040
Rieppeleon brevicaudatus	Brev01	♂	6	55	14.2	99.5	12.3	5.41	1,620	11,620
Rieppeleon brevicaudatus	Brev02	♀	2	50	13.5	101.0	14.3	4.90	1,520	9,420
Rieppeleon brevicaudatus	Brev03	♂	3	50	13.4	88.4	15.0	4.64	1,340	8,240
Rieppeleon brevicaudatus	Brev04	♀	2	45	12.6	65.9	18.7	3.60	1,090	5,120
Rieppeleon brevicaudatus	Brev05	♂	1	45	11.5	37.0	9.7	4.15	1,210	6,740
Rieppeleon brevicaudatus	Brev06	♀	1	50	12.7	56.2	13.3	4.74	1,350	9,020
Rieppeleon brevicaudatus	Brev07	♀	2	45	12.0	49.2	11.0	4.29	1,480	8,660
Trioceros cristatus	cristatus01	♀	10	108	24.3	165.5	36.7	4.70	763	5,220
Trioceros hoehnelii	hoehnelii01	♂	7	88	23.0	203.3	44.3	4.06	757	3,480
Trioceros hoehnelii	hoehnelii02	♀	2	78	18.2	169.4	48.4	4.28	757	4,500
Trioceros hoehnelii	hoehnelii03	♀	3	75	17.9	110.7	31.0	4.03	751	4,240
Trioceros jacksonii xantholophus	jacksonii01	♂	2	100	24.5	160.5	34.0	4.43	641	4,140
Trioceros johnstoni	johnstoni01	♂	3	115	25.2	193.0	47.7	4.60	608	4,000
Trioceros montium	montium01	♀	8	93	20.8	175.8	39.3	4.21	670	3,920
Trioceros montium	montium02	♂	5	107	23.7	159.0	33.3	4.49	701	4,560

The total number of feedings for each individual is given as *n*. Reported performance values represent the highest performance across all collected feedings for each individual.

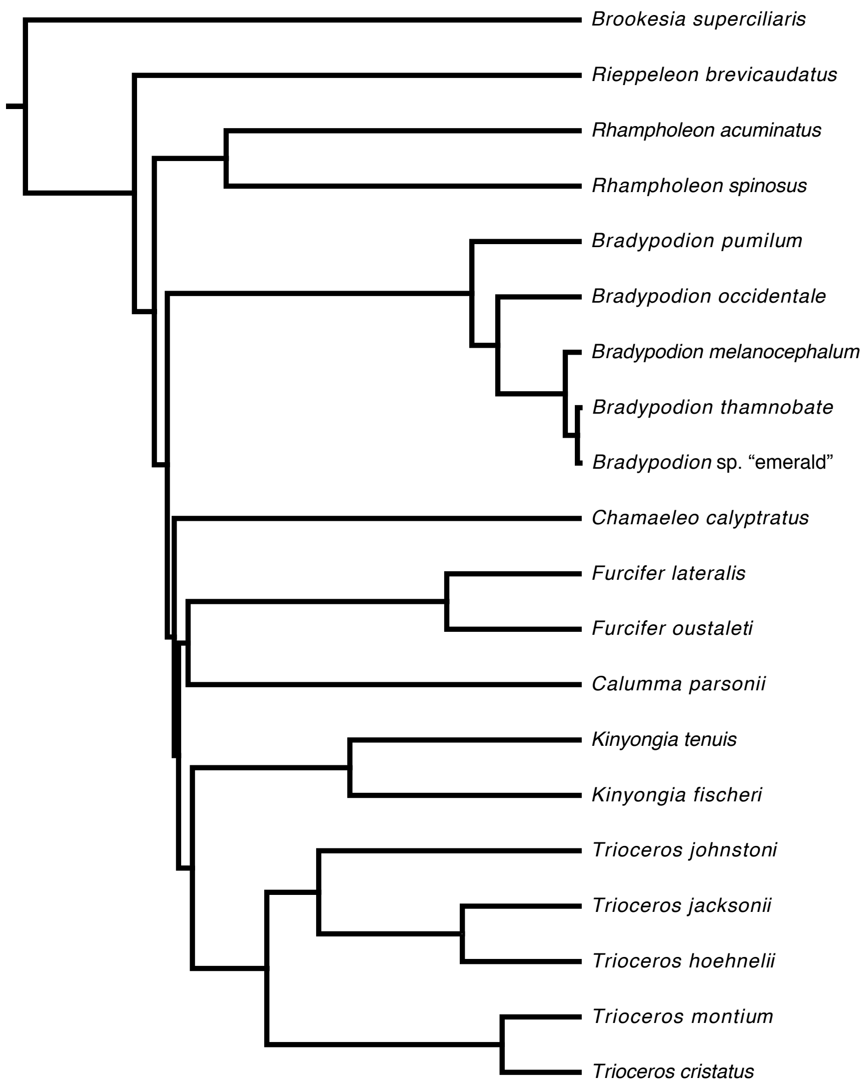

Supplementary Fig. S1. Chronogram estimating the relationships among species used in this study for phylogenetic comparative methods. Phylogenetic hypothesis based on a pruned chronogram from Tolley et al.[49] with the addition of *Bradypodion* sp. “emerald” as a sister taxon to *B. thamnobates* and assigned branch lengths close to zero based on other published studies incorporating this as yet undescribed taxon[51-54].

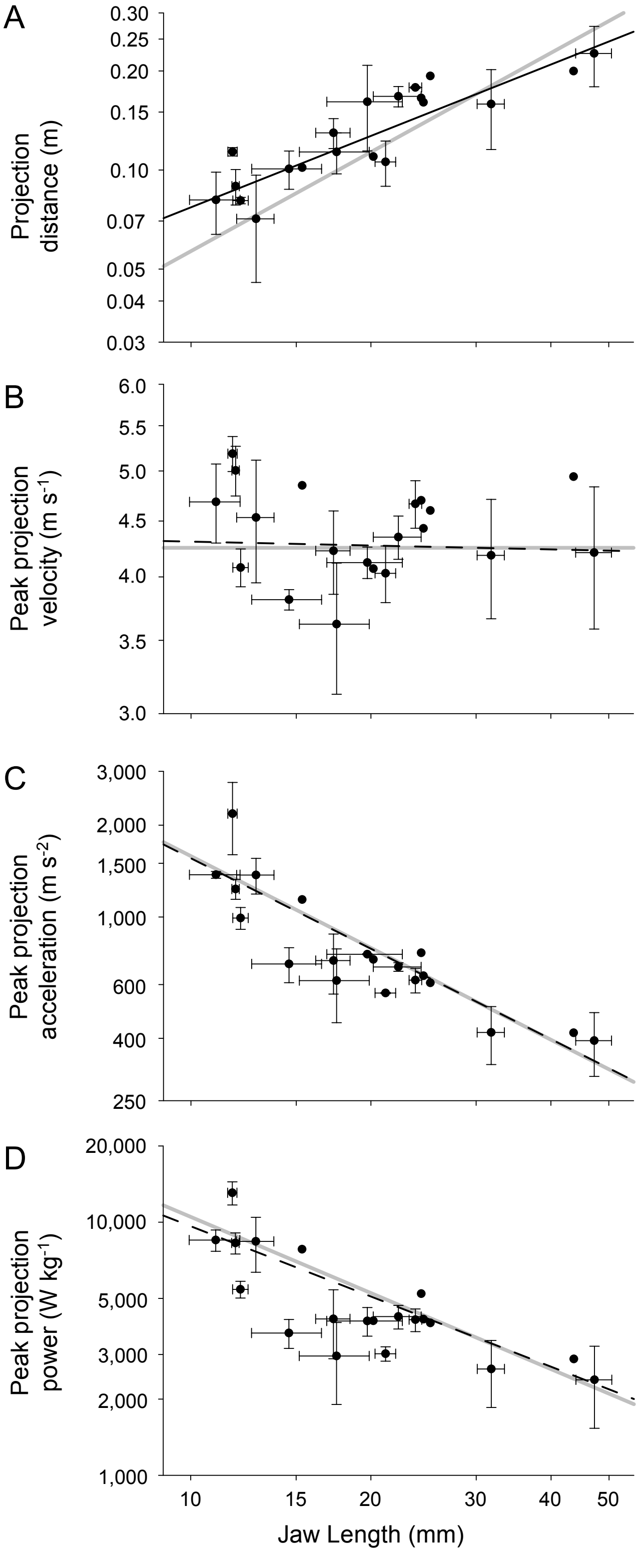
A
0.30
0.25
0.20
0.15
0.10
0.07
0.05
0.04
0.03
Projection distance (m)
B
6.0
5.5
5.0
4.5
4.0
3.5
3.0
Peak projection velocity (m s-1)
C
3,000
2,000
1,500
1,000
600
400
250
Peak projection acceleration (m s-2)
D
20,000
10,000
5,000
3,000
2,000
1,000
Peak projection power (W kg-1)
10
15
20
30
40
50
Jaw Length (mm)

Supplementary Fig. S2. Scaling relationships among species for (A) peak projection distance, (B) peak projection velocity, (C) peak projection acceleration and (D) peak mass-specific power output with respect to jaw length. Graphs depict raw species averages and standard deviations of maximal performance for individuals on log axes. Solid light gray lines represent isometric slope. Dark gray lines represent observed scaling relationships among species. Solid dark gray lines represent observed scaling relationships significantly different from that expected by isometry (i.e., expected slope falls outside the 95% confidence interval around the observed slope). Dashed dark gray lines represent observed scaling relationships not significantly different from that expected by isometry.

References

49. Tolley, K. A., Townsend, T. M. & Vences, M. Large-scale phylogeny of chameleons suggests African origins and Eocene diversification. *Proc. R. Soc. B* **280**, 20130184 (2013).

51. da Silva, J. M. & Tolley, K. A. Ecomorphological variation and sexual dimorphism in a recent radiation of dwarf chameleons (*Bradypodion*). *Biol. J. Linn. Soc.* **109**, 113-130 (2013).

52. da Silva, J. M., Herrel, A., Measey, G. J. & Tolley, K. A. Sexual dimorphism in bite performance drives morphological variation in chameleons. *PLoS ONE* **9**, e86846 (2014).

53. Tolley, K. A., Tilbury, C. R., Branch, W. R. & Matthee, C. A. Phylogenetics of the southern African dwarf chameleons, *Bradypodion* (Squamata: Chamaeleonidae). *Mol. Phylogenet. Evol.* **30**, 534-565 (2004).

54. Tolley, K. A., Chase, B. M. & Forest, F. Speciation and radiations track climate transitions since the Miocene Climatic Optimum: a case study of southern African chameleons. *J. Biogeogr.* **35**, 1402-1414 (2008).
